# Dopamine D2 receptor-mediated neuroprotection in a G2019S Lrrk2 genetic model of Parkinson’s disease

**DOI:** 10.1038/s41419-017-0221-2

**Published:** 2018-02-12

**Authors:** Alessandro Tozzi, Michela Tantucci, Saverio Marchi, Petra Mazzocchetti, Michele Morari, Paolo Pinton, Andrea Mancini, Paolo Calabresi

**Affiliations:** 1 Santa Lucia Foundation IRCCS, Rome, Italy; 2 Department of Experimental Medicine, Section of Physiology and Biochemistry, University of Perugia, Perugia, Italy; 3 Neurological clinic, Department of Medicine, University of Perugia, Santa Maria della Misericordia Hospital, Perugia, Italy; 4 Department of Morphology, Surgery and Experimental Medicine, University of Ferrara, Ferrara, Italy; 5 Department of Medical Sciences, University of Ferrara, Ferrara, Italy

## Abstract

Parkinson’s disease (PD) is a neurodegenerative disorder in which genetic and environmental factors synergistically lead to loss of midbrain dopamine (DA) neurons. Mutation of leucine-rich repeated kinase2 (Lrrk2) genes is responsible for the majority of inherited familial cases of PD and can also be found in sporadic cases. The pathophysiological role of this kinase has to be fully understood yet. Hyperactivation of Lrrk2 kinase domain might represent a predisposing factor for both enhanced striatal glutamatergic release and mitochondrial vulnerability to environmental factors that are observed in PD. To investigate possible alterations of striatal susceptibility to mitochondrial dysfunction, we performed electrophysiological recordings from the nucleus striatum of a G2019S Lrrk2 mouse model of PD, as well as molecular and morphological analyses of G2019S Lrrk2-expressing SH-SY5Y neuroblastoma cells. In G2019S mice, we found reduced striatal DA levels, according to the hypothesis of alteration of dopaminergic transmission, and increased loss of field potential induced by the mitochondrial complex I inhibitor rotenone. This detrimental effect is reversed by the D2 DA receptor agonist quinpirole *via* the inhibition of the cAMP/PKA intracellular pathway. Analysis of mitochondrial functions in G2019S Lrrk2-expressing SH-SY5Y cells revealed strong rotenone-induced oxidative stress characterized by reduced Ca^2+^ buffering capability and ATP synthesis, production of reactive oxygen species, and increased mitochondrial fragmentation. Importantly, quinpirole was able to prevent all these changes. We suggest that the G2019S-Lrrk2 mutation is a predisposing factor for enhanced striatal susceptibility to mitochondrial dysfunction induced by exposure to mitochondrial environmental toxins and that the D2 receptor stimulation is neuroprotective on mitochondrial function, *via* the inhibition of cAMP/PKA intracellular pathway. We suggest new possible neuroprotective strategies for patients carrying this genetic alteration based on drugs specifically targeting Lrrk2 kinase domain and mitochondrial functionality.

## Introduction

Leucine-rich repeat kinase 2 (Lrrk2) is a large protein with a GTPase, kinase, and scaffolding domain, implicated in a wide range of diseases^1^. Among neurodegenerative diseases, mutations in Lrrk2 are recognized as genetic risk factors for familial Parkinson’s disease (PD) and may also represent causal factors in the more common sporadic form of PD^2^. Lrrk2 is expressed in nigral and striatal neurons suggesting a role of this protein in PD degenerative processes^3^. The physiological and pathological role of Lrrk2 has not been elucidated yet; however, evidence that a gain of function of mutated kinase activity affects synaptic transmission and neuronal viability has been reported^4^.

The coincidence of environmental toxicity and genetic factors may participate in PD pathogenesis and progression^5^. Exposure to chronic pesticides, such as the mitochondrial complex I inhibitor rotenone, may in fact enhance the possibility to develop PD^6,7^. Accordingly, this neurotoxin has been extensively used to model PD^8–10^.

Lrrk2 mutations cause mitochondrial impairment and neurodegeneration, suggesting a primary role of mitochondrial activity in Lrrk2-dependent apoptotic signaling^11^. Exposure to rotenone may in fact speed up neurodegenerative processes triggered by Lrrk2 mutations by directly affecting mitochondrial homeostasis^12^. Mitochondrial functions (i.e., oxidative phosphorylation, Ca^2+^ buffering, and control of reactive radical species) are inhibited by toxins targeting mitochondrial complexes, and may lead to irreversible neuronal membrane changes, molecular alterations, and possibly to cell death.

Among monogenic forms of PD, the G2019S is the more frequent Lrrk2 mutation, a genetic alteration conferring gain of function of kinase site of the protein^4^. While the exact role of this specific Lrrk2 mutation in PD is not known yet, recent findings in PD patients have provided evidence that the G2019S Lrrk2 mutation is linked to impaired mitochondrial morphology and function^13^. However, whether impaired mitochondrial function is due to increased Lrrk2 kinase activity or other mechanisms is still unknown.

Within the nucleus striatum, DA neurotransmission is impaired in rodent models expressing Lrrk2 G2019S mutations, in the absence of neuronal loss^4,11,14–22^ and dopaminergic neurons show selective vulnerability to rotenone in a *C*. *elegans* model with Lrrk2 G2019S mutation^23^. These data suggest that Lrrk2 plays an important role in modulating the response to mitochondrial inhibition and raises the possibility that mutations in Lrrk2 selectively enhance the vulnerability of dopaminergic neurons to a stressor associated with PD.

Translation of basic research to disease-modifying therapies for PD has been unsuccessful so far. While L-dopa is still the pharmacological gold standard for PD, among other symptomatic treatments for motor symptoms of PD, DA receptor agonists had been found to be promising, although their use in the clinic is still nondefinitive. Among neuroprotective strategies to limit the PD progression, D2 DA receptor agonists are often a part of pharmacological therapy for early PD^24–26^ and could protect neurons of both the nucleus striatum and the substantia nigra by a variety of actions including the modulation of mitochondrial function. Thus, neuroprotection of cortico- and nigrostriatal circuits is a direct consequence of the restorative dopaminergic strategies. Accordingly, the D2 DA receptor agonist quinpirole was found to exert neuroprotective effects on the early alterations of mitochondrial morphology in *D*. *melanogaster* dopaminergic neurons treated with rotenone^27^.

In order to gain insights on the relation among the G2019S Lrrk2 mutation, mitochondrial function, and neuronal viability, in this study, we assessed electrophysiological recordings from the nucleus striatum of a G2019S Lrrk2 mouse model, as well as molecular and morphological analyses of G2019S Lrrk2-expressing SH-SY5Y neuroblastoma cells. We determined the effect of exposure to the neurotoxin rotenone of striatal slices and cells expressing the G2019S Lrrk2 mutation and established a major role for the D2 DA receptor agonist quinpirole in reversing the increased sensitivity of these cells to the environmental mitochondrial toxin rotenone.

## Results

### G2019S KI Lrrk2 mice present enhanced striatal sensitivity to mitochondrial complex I inhibition by rotenone

**Fig. 1:**
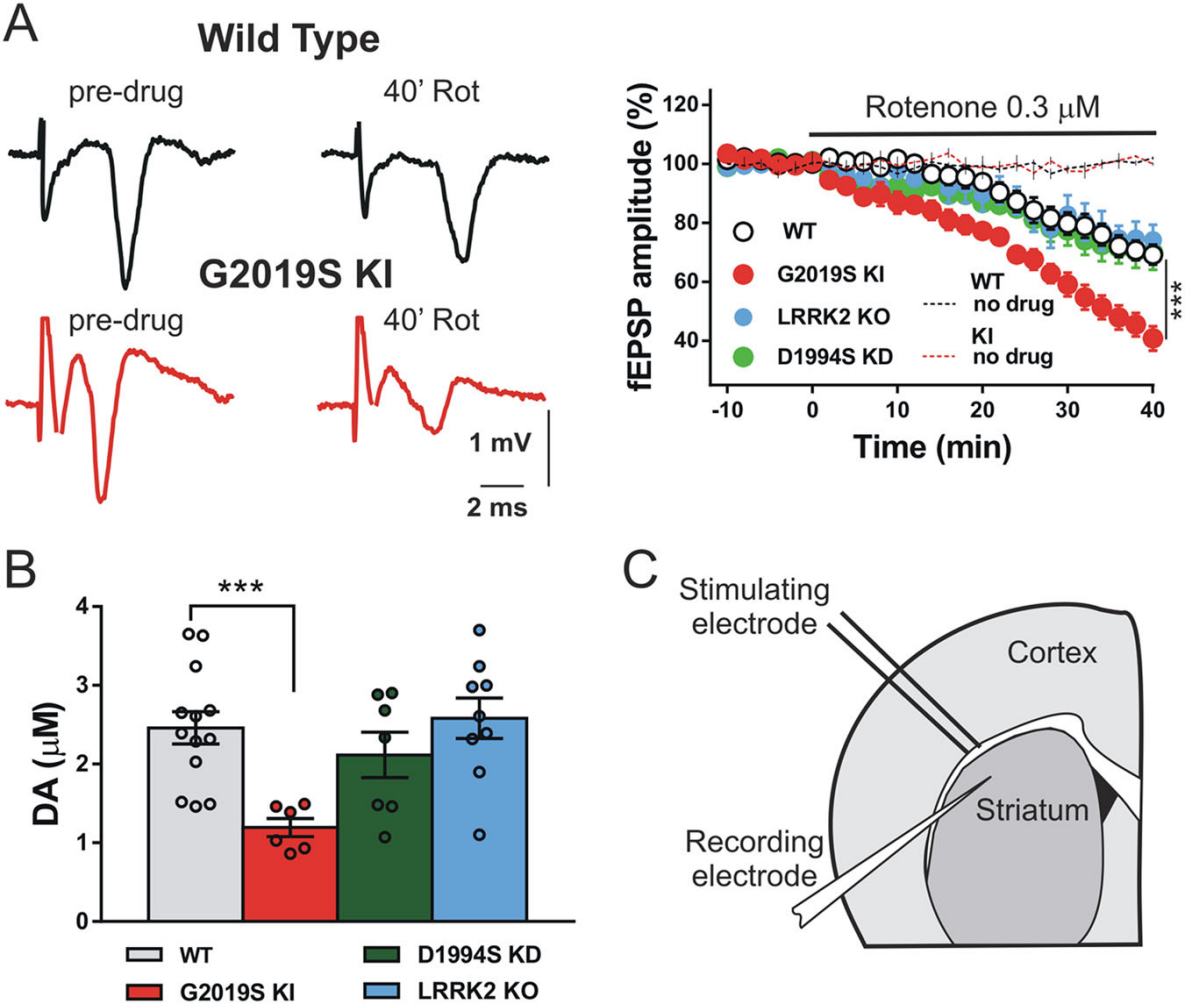
Enhanced striatal sensitivity of the field potential amplitude to rotenone and reduced dopamine levels in G2019S Lrrk2 mice. **a** Representative pairs of traces and time-course graph showing the fEPSP amplitudes before (predrug) and after the application of 0.3 µM rotenone applied for 40 min on striatal slices of wild-type (WT) (paired *t* test, *t*_(13)_ = 9.0, *p* < 0.001), G2019S KI (paired *t* test, *t*_(14)_ = 14.4, *p* < 0.001), Lrrk2 KO (*t* test *t*_(5)_ = 5.30, *p* < 0.01), and D1994S KD mice (paired *t* test, *t*_(13)_ = 5.49, *p* < 0.001); (G2019S KI vs. WT, two-way ANOVA, group main factor, *F*_(1,27)_ = 21.15, *p* < 0.001), (one-way ANOVA, *F*_(3,45)_ = 10.9, post hoc tests, *p* < 0.001). Dashed lines showing the time course of the fEPSP amplitudes recorded for 60 min in slices of WT and G2019S KI mice in the absence of rotenone. **b** Histogram showing DA concentrations measured by constant potential amperometry in striatal slices of WT, G2019S KI, Lrrk2 KO, and D1994S KD mice (one-way ANOVA, F_(3,31)_ = 5.63, *p* < 0.01; post hoc tests, KI vs. WT, *t*_(31)_ = 3.66, *p* < 0.01, KD vs. WT, *p* > 0.05, KO vs. WT, *p* > 0.05). **c** Drawing of a coronal section of a mouse corticostriatal slice showing the position of the recording and stimulating electrodes. ***p < 0.001

Coincidence of environmental and genetic factors may participate in PD pathogenesis and the toxic effects related to Lrrl2 mutations might be enhanced by neurotoxic compounds targeting mitochondria, resulting in synaptic and neuronal damage. We explored the effect of rotenone, a mitochondrial complex I inhibitor known to induce progressive striatal neurodegeneration with a dose-dependent effect^28–30^, on striatal slices of mice expressing the G2019S Lrrk2 mutation (KI). Striatal fEPSPs were recorded from slices of KI and WT mice for 10 min to obtain a stable baseline and then for further 40 min in the presence of 0.3 μM rotenone. We found that while fEPSP amplitude was stable for up to 60 min from the onset of the recording in slices of WT (*n* = 3) and KI (*n* = 3) mice, rotenone induced a slow, progressive reduction of the fEPSP amplitude either in slices of WT mice or in those of KI mice, suggesting alteration of neurotransmission, potentially reflecting neuronal death. In fact, rotenone reduced the fEPSP amplitude after 40 min of application by 30.8 ± 3.4% in slices of WT mice (*n* = 14) and by 59.2 ± 4.2% in those of KI animals (*n* = 15) (Fig. 1a). These data suggest a particularly detrimental effect of Lrrk2 hyperactivity on neuronal sensitivity, reflecting increased striatal mitochondrial dysfunction in mice carrying the G2019S mutation.

To confirm this hypothesis, we analyzed mitochondrial sensitivity to rotenone in striatal slices from two other Lrrk2-related genetic models, D1994S KD and Lrrk2 KO mice, that presented a disrupted kinase site or the absence of Lrrk2 protein, respectively. fEPSP amplitudes in the presence of rotenone were reduced by 30 ± 5.8% in slices of KD mice (*n* = 14) and by 25.8 ± 5.1% in slices of KO animals (*n* = 6) showing no significant differences with reductions of the fEPSP observed in WT mice (Fig. 1a). After 40 min of rotenone application, the progressive reduction of the fEPSP amplitude was significantly different in slices of KI versus KD and KO animals, suggesting that the G2019S Lrrk2 mutation is responsible for the increased toxicity exerted by rotenone in the striatum (Fig. 1a).

### Striatal dopamine levels are reduced in G2019S Lrrk2 mice

Altered dopaminergic signaling associated to reduced DA levels had been reported in Lrrk2 models of PD^4,11,14,16–22^. Thus, we utilized constant potential amperometry to explore possible changes in DA concentration in the striatum of the G2019S mouse model of PD. Electrical stimulation of the slice induced glutamatergic synaptic transmission and release of DA from dopaminergic terminals in the dorsolateral striatum. Peaks amplitudes of DA were measured by amperometric measures in slices of G2019S KI, D1994S KD, KO, and WT mice (Fig. 1b). The calculated DA concentration in slices of WT mice was 2.46 ± 0.2 μM and statistical analysis of the current peak amplitudes revealed differences among genotypes (one-way ANOVA, *p* < 0.01). DA concentrations were 1.19 ± 0.12 μM in slices of KI animals (*n* = 13), 2.12 ± 0.23 μM in those of KD (*n* = 7), and 2.58 ± 0.26 μM in KO mice (*n* = 9) (Fig. 1b).

These data demonstrate that the G2019S Lrrk2 mutation is associated to reduced levels of striatal DA release achieved by electrical stimulation of dopaminergic fibers, confirming that in the dorsal striatum of this genetic model of PD, the dopaminergic signaling is altered.

### Dopamine D2 receptor stimulation normalizes neuronal sensitivity to mitochondrial complex I inhibition in G2019S Lrrk2 mice

**Fig. 2:**
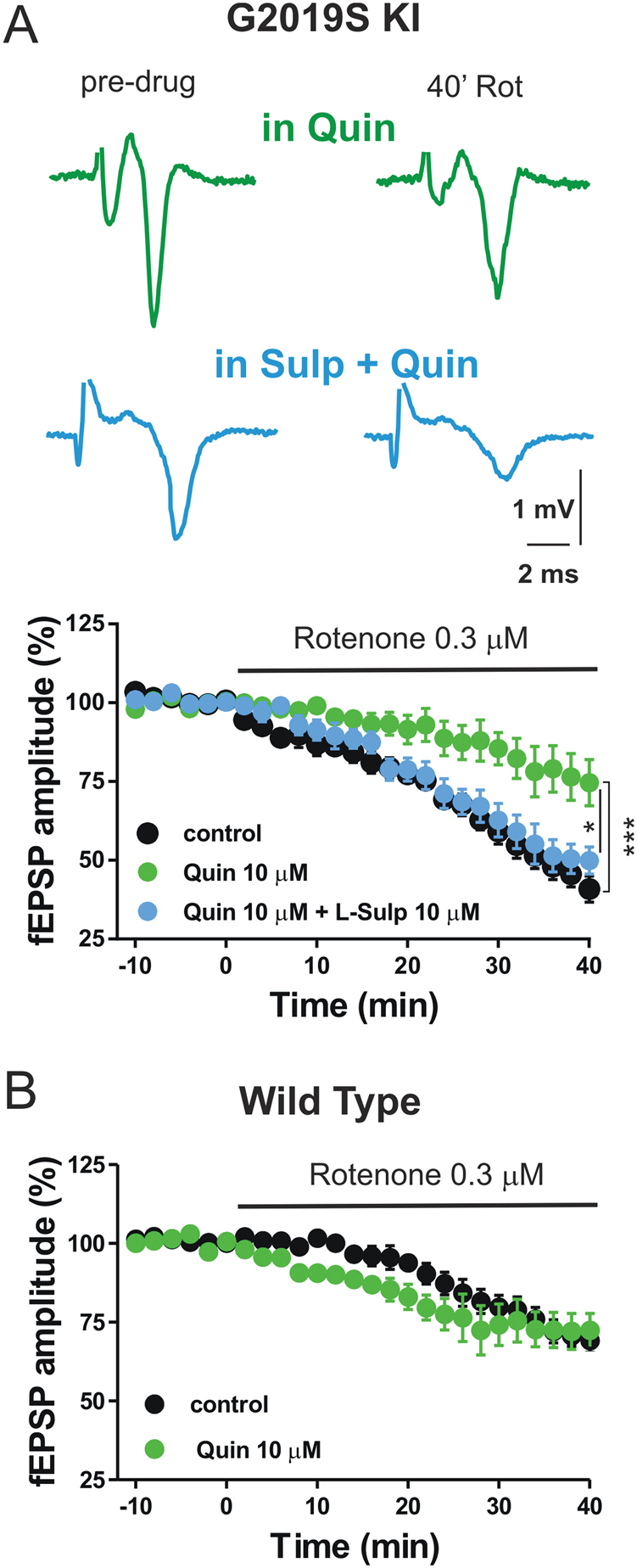
Effect of quinpirole on the field potential amplitude in striatal slices of G2019S Lrrk2 mice treated with rotenone. **a** Example traces of fEPSPs recorded before (predrug) and after the application of 0.3 µM rotenone either in the presence of 10 µM quinpirole (top traces) or quinpirole plus 10 µM l-sulpiride (bottom traces). Graph showing the time course of the fEPSP amplitude measured in striatal slices of G2019S KI mice in control conditions, in the presence of quinpirole and quinpirole plus L-sulpiride (one-way ANOVA, *F*_(2, 24)_ = 10.46, post hoc test, Rot vs. Rot + Quin, *t*_(24)_ = 4.57, *p* < 0.001, Rot + Quin vs. Rot + Quin + Sulp, *t*_(24)_ = 2.62, *p* < 0.05). **b** Time course of the fEPSP amplitude measured in striatal slices of WT mice in control conditions and in the presence of quinpirole (Rot vs. Rot + Quin, unpaired *t* test, *p* > 0.05). *p < 0.05, ***p < 0.001

We hypothesized a relation between the reduced striatal DA levels in KI mice and neuronal sensitivity to rotenone. Thus, we attempted a restorative strategy by exposing striatal slices of Lrrk2 KI mice to the D2 receptor agonist quinpirole to address its putative neuroprotective role. Striatal fEPSPs were recorded in slices of KI and WT mice in the presence of 10 μM quinpirole. After acquiring a stable baseline for 10 min, 0.3 μM rotenone was applied for further 40 min. Exposure of the slices to quinpirole significantly reduced the effect of rotenone on the fEPSP amplitude in G2019S KI mice (*n* = 7), suggesting a neuroprotective effect triggered by D2 DA receptor stimulation (Fig. 2a). We confirmed the specificity of quinpirole on D2 receptors by recording fEPSPs in slices of KI mice in the continuous presence of 10 μM L-sulpiride (*n* = 5), a D2 receptor antagonist that we coapplied with quinpirole. In these conditions, quinpirole was not able to reduce neuronal sensitivity to mitochondrial impairment in this genetic model of PD, further suggesting that the neuroprotective effect of quinpirole on rotenone-induced reduction of fEPSP amplitude involves the stimulation of D2 receptors (Fig. 2a). Conversely, we did not observe neuroprotective effects of quinpirole in slices of WT mice treated with rotenone. In fact, in these animals, 40 min of rotenone application produced a similar reduction of the fEPSP amplitude in the presence of quinpirole or in the absence of this drug (Fig. 2b).

### The protective effect of DA D2R activation is mediated by the inhibition of cAMP/PKA pathway

**Fig. 3:**
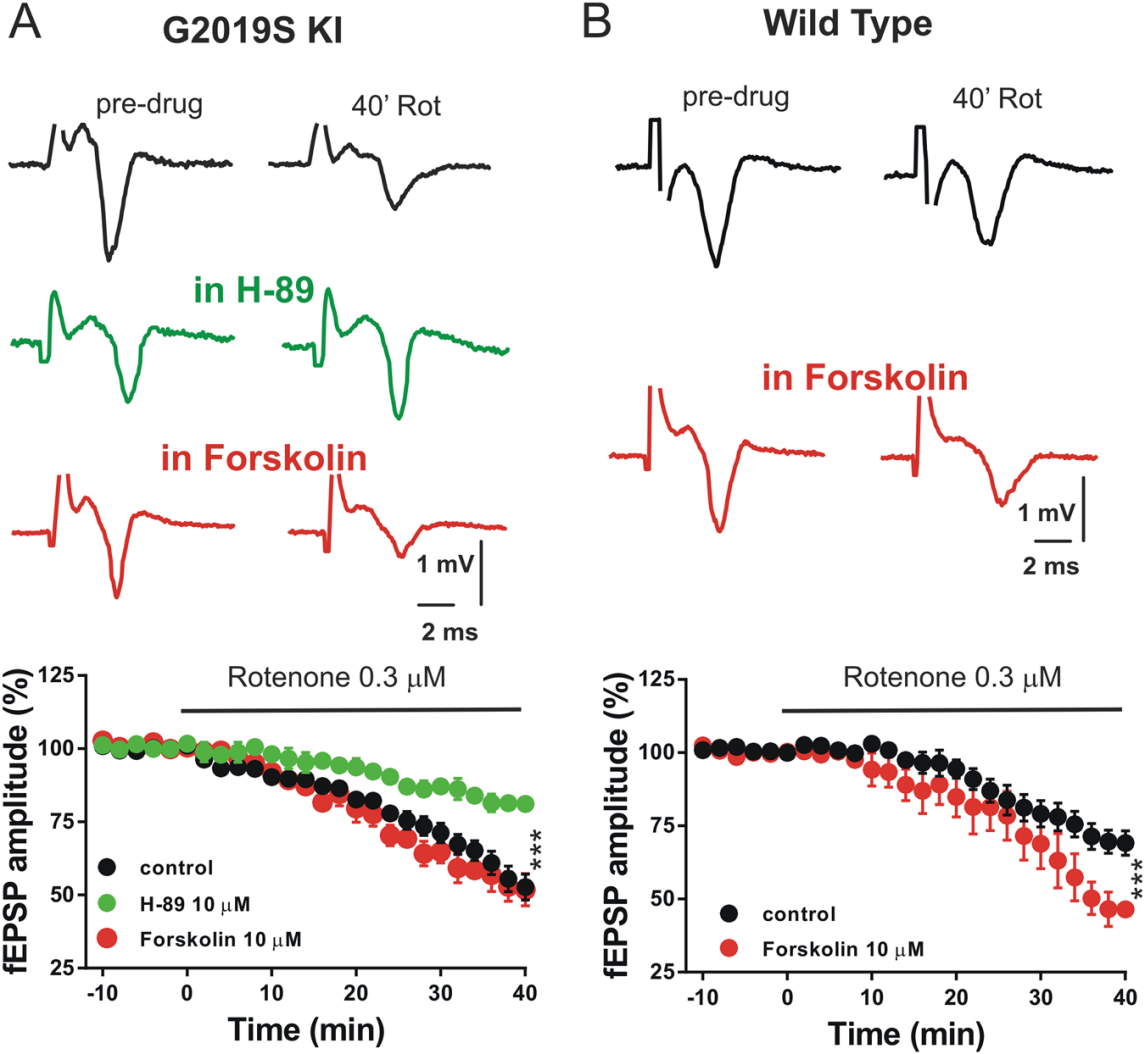
Quinpirole prevents the rotenone-induced fEPSP reduction in striatal slices of G2019S Lrrk2 mice by inhibition of the cAMP/PKA pathway. **a** Example traces and time-course graph of fEPSP amplitudes recorded in striatal slices of G2019S KI mice before (predrug) and after the application of 0.3 µM rotenone in control conditions, in the presence of 10 µM of the PKA inhibitor H-89 or 10 µM of the cAMP enhancer forskolin (one-way ANOVA, *F*_(2,21)_ = 9.37, post hoc test, Rot vs. Rot + H-89, *t*_(21)_ = 4.15, *p* < 0.01, Rot vs. Rot + Forskolin, *p* > 0.05). **b** Representative traces and time-course graph of fEPSP amplitude measured in slices of WT mice before and after the application of 0.3 µM rotenone in control conditions or in the presence of forskolin (unpaired *t* test, *t*_(13)_ = 3.16, *p* < 0.01). ***p < 0.001

The activation of D2 DA receptor inhibits intracellular Gi-protein-coupled adenylyl cyclase activity and cAMP-dependent PKA phosphorylation in striatal spiny projection neurons^31^. To confirm the involvement of this pathway in the protective effect of quinpirole on rotenone toxicity in G2019S KI mice, we directly modulated the cAMP/PKA targets exposing corticostriatal slices to 10 μM of the PKA inhibitor H-89, mimicking the D2 DA receptor stimulation, or to 10 μM forskolin, an enhancer of intracellular cAMP levels. We found that H-89, applied for 1 h, significantly reduced the detrimental effect of rotenone on the fEPSP amplitude, mimicking the protective effect exerted by quinpirole. Conversely, exposure of the slices to 10 μM forskolin did not change the toxic effect of rotenone on the fEPSP amplitude (Fig. 3a). Interestingly, in WT mice, exposure of the slices to forskolin enhanced the detrimental effect of rotenone (Fig. 3b), similarly to what was observed in slices of KI mice treated with rotenone (Fig. 1a).

These results suggest that the neuroprotective effect of quinpirole on mitochondrial complex I impairment in G2019S KI mice is mediated by inhibition of the cAMP/PKA intracellular pathway.

### Quinpirole restores mitochondrial calcium accumulation in G2019S Lrrk2-expressing cells exposed to rotenone

**Fig. 4:**
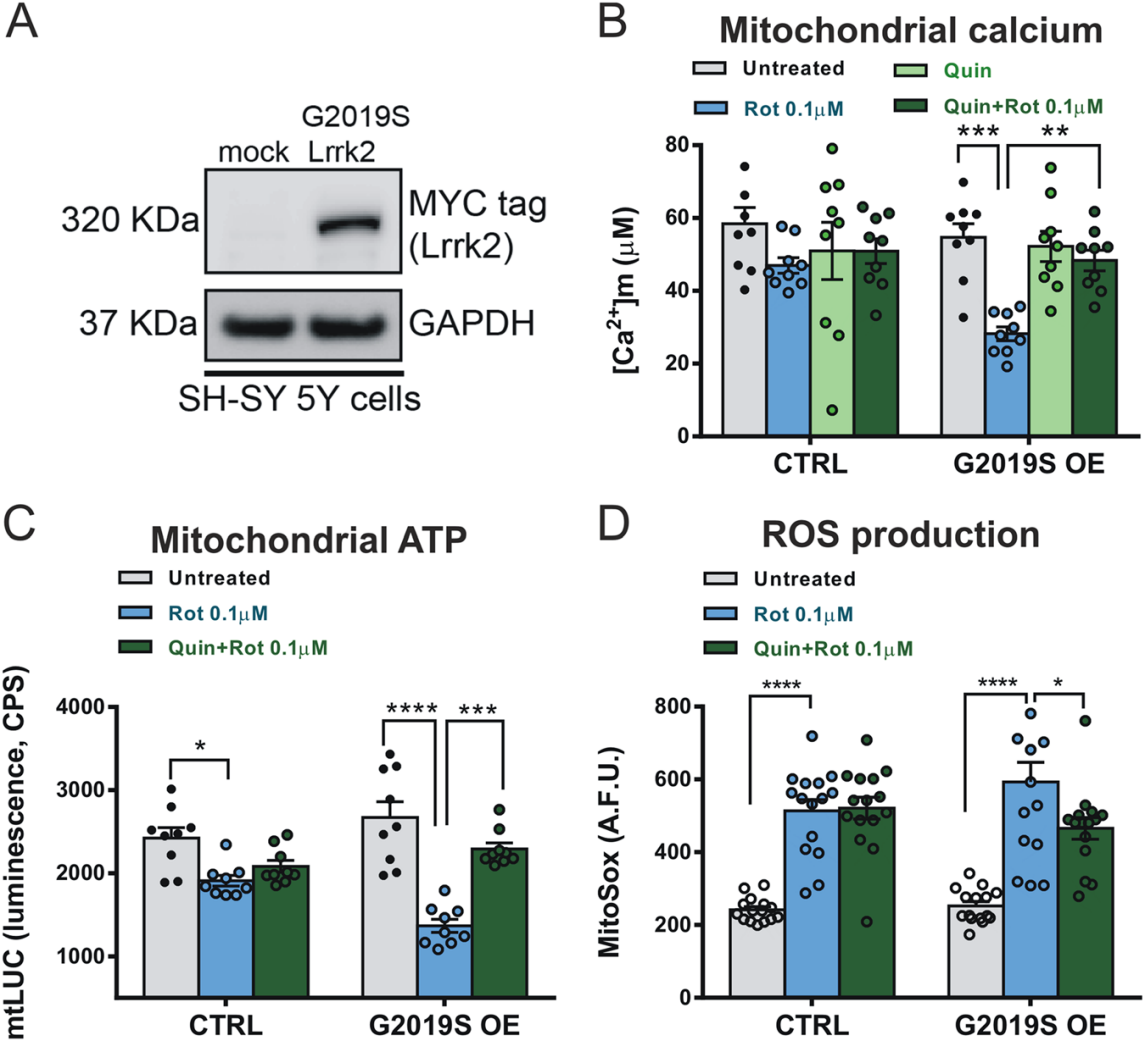
Effect of quinpirole on rotenone-induced reduction of mitochondrial Ca^2+^ uptake, ATP synthesis, and ROS production in G2019S Lrrk2-expressing SH-SY5Y cells. **a** Western blot analysis of G2019S Lrrk2 mutant expression in SH-SY5Y cells transfected with empty vector (mock) or MYC-tagged G2019S Lrrk2 construct. **b** Mitochondrial Ca^2+^ uptake in control (CTRL) and G2019S-overexpressing SH-SY5Y cells (G2019S OE) incubated with 0.1 µM rotenone, 10 µM quinpirole, or rotenone plus quinpirole ([Ca^2+^]m, two-way ANOVA, group factor, *F*_(1,64)_ = 4.03, *p* < 0.05; treatment factor, *F*_(3,64)_ = 7.40, post hoc test, G2019S Lrrk2, untreated, *n* = 9 vs. Rot, *n* = 9, *t*_(64)_ = 4.47, *p* < 0.001), (post hoc test, G2019S Lrrk2, Rot, *n* = 9 vs. Rot + Quin, *n* = 9, *t*_(64)_ = 3.40, *p* < 0.01). **c** Histogram of the mitochondrial ATP production measured in control and G2019S-expressing cells incubated with rotenone or rotenone plus quinpirole (two-way ANOVA, interaction *F*_(2,48)_ = 8.26, *p* < 0.001, post hoc tests, control untreated vs. Rot, *p* < 0.05; G2019S Lrrk2 untreated vs. Rot, *p* < 0.001); (G2019S-expressing cells, post hoc test, Rot vs. Rot + Quin, *p* < 0.001). **d** Histogram of the mitochondrial ROS production measured in control and G2019S-expressing cells incubated with rotenone or rotenone plus quinpirole (two-way ANOVA, *F*_(2,84)_ = 54.62, post hoc tests, control: untreated vs. Rot, *t*_(84)_ = 6.19, *p* < 0.001; G2019S Lrrk2: untreated vs. Rot, *t*_(84)_ = 7.75, *p* < 0.001, Quinp + Rot vs. Rot, *t*_(84)_ = 2.91, *p* < 0.05). **p < 0.01, ***p < 0.001. *n* = 3 independent experiments

We subsequently performed imaging experiments to evaluate parameters linked to mitochondrial activity, such as mitochondrial calcium changes, ATP synthesis, ROS production, and mitochondrial morphology, in control and G2019S Lrrk2-overexpressing SH-SY5Y cells. Thus, to further characterize the neuroprotective effects of quinpirole on mitochondrial function in the presence of the G2019S Lrrk2 mutation, we first used an aequorin-based approach^32^ that allowed us to measure the mitochondrial Ca^2+^ concentration ([Ca^2+^]m) following the stimulation with 500 μM carbachol, a cholinergic agonist coupled to the generation of inositol 1,4,5-trisphosphate receptor (IP3) and the release of Ca^2+^ from the endoplasmic reticulum. SH-SY5Y neuroblastoma cell lines were cotransfected with empty vector and mitochondrial-targeted aequorin (mtAEQ) Ca^2+^ probe (control) or G2019S Lrrk2 plasmid together with mtAEQ (G2019S KI). G2019S Lrrk2-expressing SH-SY5Y cells displayed higher susceptibility to low doses of rotenone (0.1 μM for 30′) compared to that of untransfected cells (Fig. 4b). Importantly, pretreatment with quinpirole in this transgenic line abolished the reduction of mitochondrial Ca^2+^ uptake induced by rotenone (Fig. 4b). Notably, quinpirole alone (*n* = 9) did not alter the mitochondrial Ca^2+^ accumulation (Fig. 4b, *p* > 0.05), suggesting that it did not exclusively act on Ca^2+^ machinery but it might possess a protective role on the whole mitochondrial homeostasis.

The effects of both rotenone and quinpirole were selective for the mitochondrial Ca^2+^, since measurements of cytosolic Ca^2+^ concentration ([Ca^2+^]c) using a cytosolic aequorin Ca^2+^ tool (cytAEQ) revealed no significant differences both in untreated control and G2019S SH-SY5Y neuroblastoma cells, as well as in the presence of rotenone or quinpirole plus rotenone (data not shown).

### Quinpirole restores mitochondrial ATP synthesis and ROS production in G2019S Lrrk2-expressing cells exposed to rotenone

Maintenance of cellular homeostasis strongly relies on energy supply by ATP synthesis and preservation of reduced levels of reactive oxygen species (ROS). Since ATP synthesis directly reflects mitochondrial function, we measured mitochondrial ATP production in both control and G2019S Lrrk2-overexpressing cells using a targeted luciferase-based ATP probe (mtLUC). We found a significant effect of rotenone on ATP production in both control and G2019S Lrrk2 cells (Fig. 4c). Interestingly, the pretreatment of SH-SY5Y cells with 10 µM quinpirole prevented the rotenone-dependent reduction of ATP levels only in G2019S-expressing cells, while it had no effect on ATP levels of control cells (Fig. 4c). Thus, expression of G2019S Lrrk2 in SH-SY5Y cells conferred strong sensitivity to rotenone, as revealed by altered mitochondrial calcium-buffering properties and reduced ATP production.

We subsequently tested whether the augmented mitochondrial sensitivity of G2019S Lrrk2 cells to rotenone could directly reflect increased ROS levels and oxidative stress. Measurements of mitochondrial superoxide anion levels using the MitoSox fluorescent probe revealed that rotenone induced production of a high amount of ROS, in both control and G2019S Lrrk2-expressing cells (Fig. 4d). Quinpirole pretreatment, however, significantly reduced the superoxide levels produced by rotenone in G2019S cells, while in control cells, it was ineffective.

Overall, these findings suggest that rotenone directly affects ATP and ROS production and that quinpirole is a valuable protective agent able to prevent changes to mitochondrial energetic balance.

### Quinpirole prevents rotenone-induced mitochondrial fragmentation in G2019S Lrrk2-expressing cells

**Fig. 5:**
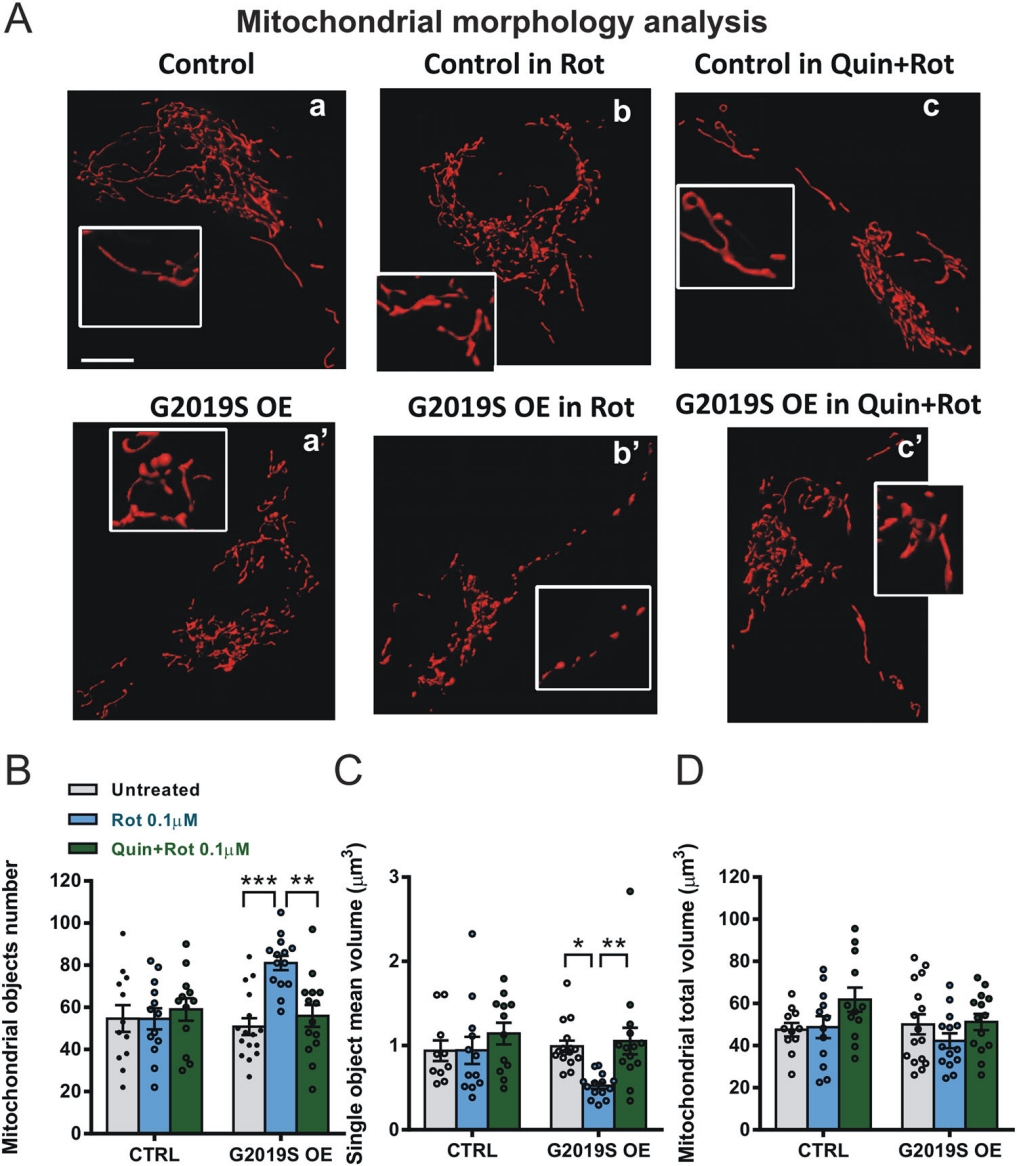
Effect of rotenone and quinpirole on mitochondrial morphology in G2019S-expressing SH-SY5Y cells. **a** Representative images of mitochondria of control SH-SY5Y cells and G2019S-expressing cells (G2019S OE) untreated (a, a’), treated with rotenone (b, b′), or treated with rotenone plus quinpirole (c, c′). Scale bar, 5 µm. **b**–**d** Histograms showing the mitochondrial object number (**b**) the mean volume of single objects (**c**) and the total mitochondrial volume (**d**) calculated in control and G2019S-overexpressing SH-SY5Y cells, treated or not with rotenone or with rotenone plus quinpirole (two-way ANOVA, treatment factor, object number: *F*_(2,75)_ = 5.04, *p* < 0.01, post hoc tests: untreated vs. Rot, *t*_(75)_ = 4.63, *p* < 0.001, Rot vs. Rot + Quin, *t*_(75)_ = 3.72, *p* < 0.01; object volume: *F*_(2,72)_ = 4.90, post hoc tests, untreated vs. Rot, *t*_(72)_ = 3.02, *p* < 0.05, Rot vs. Rot + Quin, *t*_(72)_ = 3.31, *p* < 0.01; total volume: *p* > 0.05). *p < 0.05, **p < 0.01, ***p < 0.001. *n* = 3 independent experiments

Rotenone produces neurotoxicity mainly by oxidative stress^33^. However, mitochondrial fragmentation is an early event in rotenone-induced toxicity^34–36^. High levels of mitochondrial fission increase ROS production, disrupt Ca^2+^ homeostasis, and promote cell death^35^. Since blocking mitochondrial fission alleviates rotenone-mediated apoptosis in primary cortical neurons^37^, we explored whether, in G2019S-Lrrk2-expressing cells, rotenone altered cellular homeostasis by directly modifying mitochondrial morphology. We used a red fluorescent protein with mitochondrial presequence (mtDsRed) for monitoring the organelle structure. SH-SY5Y control cells displayed an interconnected three-dimensional mitochondrial network (Fig. 5Aa), which appeared to be unchanged following treatment with 0.1 μM rotenone (Fig. 5Ab,c). Conversely, G2019S Lrrk2-expressing cells showed a highly fragmented mitochondrial compartment following 0.1 μM rotenone application (Fig. 5Aa’,b’). Subsequently, we tested the effect of quinpirole on rotenone-induced mitochondrial fragmentation. Interestingly, the coapplication of rotenone plus quinpirole was able to prevent mitochondria fragmentation in G2019S Lrrk2 cells, while it had no effect in control cells (Fig. 5Ac,c’). Quantitative analysis of different morphological parameters revealed that rotenone strongly increased the number of mitochondrial objects in G2019S-transfected cells, with concomitant reduction of dimension of the single object and no alterations of the total mitochondrial volume. Moreover, the coapplication of rotenone plus quinpirole prevented the effect of rotenone on both the number and the size of mitochondrial objects (Fig. 5 b–d).

Thus, huge mitochondrial fission occurs in G2019S Lrrk2-positive cells when exposed to rotenone, and quinpirole treatment is able to preserve mitochondrial network from extensive fragmentation. Taken together, these data suggest that quinpirole protects cells from rotenone-mediated mitochondrial damage normalizing Ca^2+^ buffering, ATP synthesis, reducing mitochondrial ROS production, and preserving the physiological (i.e., filamentous) mitochondrial architecture.

## Discussion

Mutations of Lrrk2 gene represent the most common genetic mutation associated to PD, both in familial and sporadic forms^2,38^. Considering its frequency in patients affected by PD, it could represent a predisposing factor able to increase the susceptibility to toxic environmental factors concurring to disease pathogenesis and progression. In this work, we utilized a genetic G2019S Lrrk2 mouse model of PD and cells expressing the same mutation to analyze possible alterations of striatal susceptibility to oxidative stress. In this model, oxidative stress was produced by applying rotenone, an environmental toxin commonly used as organic insecticide, that has been associated with mitochondrial complex I dysfunction in PD^39^.

We obtained three novel major findings: i) the striatum of Lrrk2 KI mice is more sensitive to rotenone, since this neurotoxin strongly reduced the field potential amplitude with respect to WT mice, indicating a pronounced alteration of neurotransmission, likely reflecting neuronal suffering/death; ii) the G2019S Lrrk2 mutation is associated to enhanced rotenone-dependent alteration of mitochondrial homeostasis, as indicated by impaired Ca^2+^ buffering capability, reduced ATP synthesis, increased ROS production, and mitochondrial fragmentation; and iii) the stimulation of the D2 DA receptor by quinpirole, through the inhibition of cAMP/PKA intracellular pathway, prevented the augmented electrophysiological alterations observed in striatal slices in Lrrk2 KI mice, and protected G2019S Lrrk2 cells from rotenone-mediated mitochondrial damage.

Several animal models have been proposed to recapitulate human Lrrk2-related PD^40^. They include mice expressing the R1441G or the G2019S Lrrk2 mutation associated with slight molecular and behavioral deficits or with more pronounced age-dependent phenotypes^41–44^.

It is noteworthy that a wide variety of subtle motor and nonmotor behavioral alterations have been described in genetic Lrrk2 mouse models, depending on the expressed mutation, generally reflecting the symptoms shown in patients affected by the early phases of PD^40^. Genetic Lrrk2 models could then be crucial to investigate PD pathogenesis since its very early phases, in order to discover and test potential neuroprotective pharmacological strategies able to prevent the presentation of classical motor and cognitive symptoms affecting PD patients.

In this work, we used a G2019S Lrrk2 mouse model of PD since this mutation is the most common and frequent pathogenic mutation identified in PD worldwide, accounting for up to 1–6% of sporadic and 3–19% of familial PD^45,46^. It has been reported that these mutant Lrrk2 KI mice exhibit subtle abnormalities such as abnormal mitochondrial morphology^11^ and increased susceptibility to synaptic dysfunction even in young 3-month-old mice^18^, while they show hyperkinetic phenotype resistant to normal age-related motor decline^47^. These findings are compatible with a similar observation in humans where G2019S mutant Lrrk2 carriers showed abnormal DAT-SPECT^48,49^ and increased substantia nigra hyperechogenicity^50,51^ since the earlier premotor phase of the disease.

Here, we presented electrophysiological evidence that 6-month-old mice carrying the G2019S Lrrk2 mutation show reduced striatal DA levels and enhanced susceptibility to the neurotoxic effects produced by a submaximal dose of rotenone in the nucleus striatum. Inhibition of complex I is known to shift neurons into a state of oxidative stress triggering excitotoxic death pathways, possibly playing a crucial role in PD pathogenesis^39^. Accordingly, rotenone toxicity resulted not only from a neuronal bioenergetic defect *per se* related to reduced ATP levels but it was also primarily due to enhanced oxidative damage at which neurons were exposed^30,39^.

The potential role of the different Lrrk2 mutations in influencing neuronal vulnerability to mitochondrial impairment has been under investigation during the last few years. It has been suggested that the G2019S Lrrk2 mutation could play a significant role in favoring rotenone-induced striatal toxicity, since *C*. *elegans* expressing this specific mutation showed a greater loss of striatal dopaminergic markers (such as DAT-GFP fluorescence and DA levels) in response to rotenone exposure^23^. Moreover, Lrrk2 could directly alter mitochondrial homeostasis, since profound abnormalities in mitochondrial fission processes and morphology have been described in the striatum of mice carrying the G2019S mutation^11^. Notably, overexpression of non-mutated Lrrk2 could itself affect cell viability; thus, our results obtained in SH-SY5Y cells might reflect either the enhanced Lrrk2 level and/or the presence of the G2019S mutation. The investigation of the possible molecular pathways that link the hyperactivation of Lrrk2 kinase domain of the G2019S KI mice to the enhanced neuronal susceptibility to oxidative stress is crucial for developing neuroprotective strategies for patients carrying this specific mutation and possibly for all patients affected by the early phases of the disease. Interestingly, we show that the pharmacological activation of the D2 DA receptor is able to reduce the detrimental effect of the G2019S Lrrk2 mutation on mitochondrial susceptibility to rotenone. This protective effect was evident both with electrophysiological recordings, which showed less severe progressive reduction of the excitatory striatal transmission, and with the analysis of mitochondrial morphology, ATP production, ROS expression, and Ca^2+^ uptake obtained in SH-SY5Y cells. Notably, we observed a strong effect of quinpirole in preserving the correct morphology of the mitochondrial network (Fig. 5), supporting recent evidence obtained in *Drosophila* showing that neuroprotective therapy for PD-like pathology might be initiated before mitochondrial fragmentation^27^.

Interestingly, we did not show a similar enhanced susceptibility to mitochondrial dysfunction in mice carrying a Lrrk2 kinase-domain-inactivating mutation (KD mice) or mice lacking Lrrk2 (KO mice), suggesting that the observed effects were specifically related to sustained activation of G2019S-Lrrk2 kinase activity.

Our findings suggest that the activation of the DA D2 receptor intracellular transduction pathway is involved in modulating the complex relationship between Lrrk2 kinase activity and mitochondrial homeostasis. In order to test this hypothesis, we investigated whether the pharmacological modulation of cAMP/PKA pathway, which is inhibited by the activation of DA D2 receptor, was able to influence rotenone toxicity in G2019S Lrrk2 KI mice. Indeed, the pharmacological inhibition of PKA mimicked the protective effect exerted by the DA D2 receptor activation on mitochondrial complex I dysfunction in KI mice, while its activation through the exposure to forskolin enhanced striatal rotenone toxicity in WT mice.

The complex interaction between Lrrk2 activity and both the cAMP/PKA pathway and the dopaminergic transmission is still controversial^52^. Accordingly, while the G2019S Lrrk2 mutation has been positively associated to increased striatal membrane expression of D1 DA receptor and PKA activation^53–55^, the effect of Lrrk2 mutations on the D2 receptor is more debated, with evidence suggesting that Lrrk2 could modulate D2 DA receptor expression by influencing the intracellular receptor trafficking^55^.

Cytoplasmatic cAMP levels and PKA-dependent phosphorylation of mitochondrial proteins may play a role in mitochondrial homeostasis^56–58^. However, whether cAMP/PKA signaling exerts beneficial or detrimental effects on mitochondrial metabolism is still debated^57–59^.

In line with our findings, describing neuroprotection by downregulation of PKA activity, cAMP/PKA-dependent regulation of mitochondrial dynamics, involving fusion/fission processes, motility, and mitophagy, has been reported to exert pro-survival role for mitochondria and host cells during stressing conditions^56^. Conversely, PKA-dependent phosphorylation of the pro-apoptotic protein Bax resulted in its mitochondrial translocation leading to activation of cellular apoptotic pathways^59^. Interestingly, mitochondrial overactivity of PKA can lead to hyperphosphorylation of mitochondrial complexes enhancing ROS production, an effect that could be prevented by the PKA inhibitor H-89^60,61^.

Overall, considering these evidences and our findings, we hypothesize that within the nucleus striatum, hyperactivity of Lrrk2 kinase domain, due to the G2019S mutation, induces sustained activation of the cAMP/PKA pathway. PKA hyperactivity could in turn produce detrimental effects on mitochondrial ability to recover from stressors like rotenone, leading to enhanced neuronal suffering. Considering this hypothesis, targeting the cAMP/PKA pathway to protect neurons of PD patients from Lrrk2-induced cell disease is of particular interest. In this context, a key role is played by the DA D2 receptor, whose pharmacological activation reduces the cAMP levels and PKA activity, but the knowledge of the precise mechanism by which its activation reduces the effects of rotenone in mice requires a further more detailed molecular analysis in both physiological conditions and in Lrrk2-related PD. DA agonists such as pramipexole, ropinirole, and rotigotine are frequently used, together with L-dopa, in the clinical setting and represent a well-established symptomatic therapy for patients with PD, especially in the earlier phases of the disease^62^. Our results may thus further support the use of DA agonist in the treatment of PD symptoms.

DA is known to possibly exert toxic effects in neurons by multiple mechanisms affecting cell viability in pathological conditions such as during rotenone application^63^^,^^64^. Thus, normalization of DA signaling by D2 DA receptor stimulation in the presence of environmental toxins might be beneficial for cellular models of PD overexpressing mutated Lrrk2, similarly to the neuroprotection observed in striatal neurons expressing basal mutated Lrrk2.

In deep investigation of the complex relationship among mitochondrial activities, Lrrk2 mutations and DA D2 receptor activation will help identify specific pathogenetic hallmarks in PD useful to stratify patients who could benefit from dopaminergic therapy in order to delay disease presentation and progression.

## Materials and methods

### Mice models and ethics statement on animal use

Mutant homozygous male mice carrying either the kinase-enhancing G2019S pathogenic mutation (knock-in, KI) that genocopies the human G2019S PD-causing mutation or a kinase-inactivating point mutation D1994S (kinase-dead, KD) and mice lacking Lrrk2 completely (knockout, KO) backcrossed on a C57Bl/6 J background were generated as previously reported^47,65^. Nontransgenic male wild-type (WT) mice were littermates obtained from the respective heterozygous breeding. Colonies were obtained from the University of Ferrara, they were kept under regular lighting conditions (12 h light/dark cycle), and given food and water ad libitum. All procedures were conducted in conformity with the European Communities Council Directive of November 1986 (86/609/ECC), in accordance with protocols approved by the Animal Care and Use Committee at the Universities of Perugia and the Italian Ministry of Health (0018691-P-11/09/2014). All efforts were made to minimize the number of animals used and their suffering.

### Electrophysiology

Six-month-old G2019S KI, Lrrk2 KO, D1994S KD, and age-matched WT mice were killed by cervical dislocation. The brain was rapidly removed and coronal corticostriatal slices (250 μm) were cut in Krebs’ solution (in mmol/L: 126 NaCl, 2.5 KCl, 1.2 MgCl_2_, 1.2 NaH_2_PO_4_, 2.4 CaCl_2_, 10 glucose, and 25 NaHCO_3_) using a vibratome. The slices were maintained in Krebs’ solution, bubbled with an O_2_ 95% and CO_2_ 5% gas mixture (pH = 7.4) at room temperature. Single coronal slices including the cortex and the striatum were transferred to a recording chamber and submerged in a continuously flowing Krebs’ solution (33 °C; 2.5–3 ml/min) bubbled with a 95% O_2_–5% CO_2_ gas mixture.

Glutamatergic excitatory postsynaptic field potentials (fEPSP) were evoked every 10 s by means of a bipolar electrode connected to a stimulation unit (Grass Telefactor) and located in the white matter between the cortex and the striatum to activate glutamatergic fibers. The recording borosilicate glass electrode filled with 2 mol/L NaCl (resistance 10–15 MΩ), was placed in the dorsolateral striatum (Fig. 1c).

### Constant potential amperometry

Dopamine release was induced by electrical stimulation using the same bipolar electrode used to evoke fEPSPs and was monitored by constant potential amperometry (CPA) with a carbon fiber recording electrode (World Precision Instruments) positioned into the dorsolateral striatum (depth 100–150 μm) near the stimulating electrode^66^ (Fig. 1c). The recording electrode was connected to a potentiostat (MicroC, World Precision Instruments) to apply voltage and to measure the current. The imposed voltage between the carbon fiber electrode and the silver/AgCl pellet was 0.55 V. For stimulation, we applied a single electrical shock (30 µs, 35 V) controlled by a stimulus isolation unit (Grass S88 Stimulator; Grass Instruments) every 200 s. Electrical shock caused a rapid increase in amperometric current that generally decayed back to baseline in about 1.5 s. Signals were digitized using a Digidata acquisition system (Digidata 1322 A, Molecular Devices) coupled to a PC running pClamp 9 (Molecular Devices) and monitored for 20 min. Electrode calibration was performed at the end of each experiment in ACSF containing DA (300 nmol/L–3 μmol/L).

### Cell culture and transfection

SH-SY5Y cells were cultured in Ham’s nutrient mixture F12 (HAM’S/F12), supplemented with 10% FCS, 2 mM L-glutamine, penicillin/streptomycin, and MEM nonessential amino-acid solution, in 75-cm^2^ Falcon flasks. These cells express both splice variants of the D2 DA receptor, as well as a functional DA transporter^67^. For aequorin and luciferase experiments, cells were seeded onto 13-mm glass coverslips and allowed to grow to 75% confluence; for MitoSox measurements and mitochondrial morphology analysis, cells were seeded on a 24-mm glass coverslip in the same conditions of growth. Transfection of SH-SY5Y cells was performed with a 3:1 DNA ratio (3 μg of the indicated expression plasmids and 1 μg of the reporter plasmid), using Lipofectamine LTX, according to the manufacturer’s protocol. All measurements were performed 36 h after transfection.

### Aequorin measurements

Aequorin-based Ca^2+^ measurements have been performed as previously described^32^. Briefly, the probes used are chimeric aequorins targeted to cytosol (cytAEQ) and mitochondria (mtAEQmut). “AEQ” refers to wild-type aequorin, and “AEQmut” refers to a low-affinity D119A mutant of aequorin. For the experiments with cytAEQ and mtAEQmut, cells were incubated with 5 μM coelenterazine for 2 h in HAM’S/F12 supplemented with 1% FCS. A coverslip with transfected cells was placed in a perfused thermostated chamber located in close proximity to a low-noise photomultiplier with a built-in amplifier/discriminator. All aequorin measurements were carried out in Krebs’ solution supplemented with 1 mM CaCl_2_. An agonist (carbachol, 500 μM) was added to the same medium to evoke the increase of cytosolic Ca^2+^. The experiments were terminated by lysing cells with 100 μM digitonin in a hypotonic Ca^2+^-containing solution (10 mM CaCl_2_ in H_2_O), thus discharging the remaining aequorin pool. The output of the discriminator was captured by a Thorn EMI photon-counting board and stored in a compatible computer for further analyses. The aequorin luminescence data were calibrated off-line into [Ca^2+^] values using a computer algorithm based on the Ca^2+^ response curve of wild-type and mutant aequorins.

### Immunoblot

The analysis was performed as previously described^68^. Briefly, SH-SY5Y cells were lysed on ice for 30 min in a buffer containing 50 mM Tris-HCl, pH 7.4, 150 mM NaCl, 1% Triton X-100, 0.2% SDS, protease, and phosphatase inhibitor cocktail. The cell lysate was clarified by centrifugation and 20 μg of proteins were loaded on a Novex NuPage Bis-Tris 4–12% precast gel (Thermo Fisher Scientific). Proteins were transferred to nitrocellulose membrane, blocked with 5% milk, and then incubated overnight with primary antibodies. The primary antibodies and dilutions are as follows: GAPDH (Cell Signaling, #2118; 1:5000) and MYC tag (Abcam, ab9106; 1:2000). The revelation was assessed by specific horseradish peroxidase-labeled secondary antibodies (Thermo Fisher Scientific), followed by detection of chemiluminescence (Thermo Fisher Scientific), using ImageQuant LAS 4000 (GE Healthcare).

### ATP measurements

Luciferase-based ATP measurements have been performed as previously described^69^. Cell luminescence was measured in the same purpose-built luminometer used for aequorin measurements. The probe used is a chimeric luciferase targeted to mitochondria (mtLUC). Cells were constantly perfused with KRB buffer, supplemented with 1 mM CaCl_2_ and 20 mM D-luciferin. To evoke ATP production, cells were perfused with a solution containing 20 mM D-luciferin plus 500 μM carbachol. The amount of produced ATP has been calculated by dividing the luminescence values of the second plateau generated after the addition of the luciferin plus carbachol solution by the luminescence values of the plateau generated after the addition of the luciferin solution.

### Imaging and analysis of mitochondrial morphology

SH-SY5Y cells were seeded and transfected with mtDsRed as described in the “Results” section. Protein expression was allowed for 36 h and then cells were imaged with Nikon Swept Field Confocal, equipped with CFI Plan Apo VC60XH objective (n.a. 1.4) and an Andor DU885 EM-CCD camera, controlled by the NIS-Elements 3.2. Coverslips were placed in an incubated chamber with controlled temperature, CO_2_, and humidity and then z-stacks were acquired by 21 planes with 0.6-μm distance, to allow acquisition of the whole cell. After acquisition, images were restored with the Autoquant 3D-blind deconvolution module, installed on NIS-Elements (Nikon Instruments Inc.), using a theoretical PSF. After restoration, images were loaded in Imaris 4.0, then subtracted of background, and used to generate a threshold-based isosurface object group. From each isosurface, the number of objects and the average object volume expressed as voxel number were calculated.

### Reactive oxygen species measurements

Detection of mitochondrial superoxide was performed by using MitoSOX^TM^ Red, a live-cell-permeant fluorigenic dye [3,8-phenanthridinediamine, 5-(69-triphenylphosphoniumhexyl)-5,6-dihydro-6-phenyl], which is rapidly and selectively targeted to mitochondria, where it is oxidized by superoxide, but not by other reactive oxygen species (ROS)- or reactive nitrogen species (RNS)-generating systems, and exhibits red fluorescence. Experiments were performed according to the manufacturer’s instructions (Molecular ProbesTM, Invitrogen). Briefly, cells grown to 50–80% confluence on glass coverslips were incubated with 5 µM MitoSOX in Hank’s Buffered Salt Solution containing calcium and magnesium (HBSS), for 15 min at 37 °C in a CO_2_ incubator and then washed three times with HBSS. Microscopy imaging was performed on the confocal microscope Zeiss LSM510, using a 63 × 1.4 NA Plan-Apochromat oil-immersion objective, 514-nm excitation laser, and 580 ± 30 nm fluorescence detection range.

### Drugs

Drugs were applied by dissolving them to the desired final concentration in Krebs' solution and by switching the perfusion from control solution to drug-containing solution. Carbachol, forskolin, H-89, picrotoxin, quinpirole (Quin), rotenone (Rot), and L-sulpiride (Sulp) were purchased from Tocris-Cookson (Bristol, UK). Drugs were dissolved into the external medium or Krebs' solution and applied by switching the perfusion from control solution to drug-containing solution.

### Statistical analysis

Data analysis was performed off-line using Clampfit 10 (Molecular Devices) and GraphPad Prism 6 (GraphPad Software). Values given in the text and figures are mean ± S.E., where n represents the number of slices or coverslips used for each electrophysiological, CPA, or in vitro experiments. Changes of the evoked fEPSP amplitudes induced by drugs were expressed as a percentage of the baseline, the latter representing the normalized fEPSP mean amplitude acquired during a stable period (10 min) before drug administration. Student’s *t* test, one-way, or two-way ANOVA, followed by Bonferroni’s post hoc test, were used for statistical analysis. The significance level was established at *p* < 0.05.
